# Microbiome and Metabolomics Reveal the Effects of Different Feeding Systems on the Growth and Ruminal Development of Yaks

**DOI:** 10.3389/fmicb.2021.682989

**Published:** 2021-06-22

**Authors:** Chun Huang, Fei Ge, Xixi Yao, Xian Guo, Pengjia Bao, Xiaoming Ma, Xiaoyun Wu, Min Chu, Ping Yan, Chunnian Liang

**Affiliations:** Key Laboratory of Yak Breeding Engineering Gansu Province, Lanzhou Institute of Husbandry and Pharmaceutical Science, Chinese Academy of Agricultural Sciences, Lanzhou, China

**Keywords:** yak, feeding system, rumen, microorganism, metabolomics, growth

## Abstract

The change in the feeding system can greatly improve the growth performance of the yak (*Bos grunniens*), an important livestock species in the plateau region. Here, we comprehensively compared the effects of different feeding systems on the growth performance and ruminal development of yaks, and investigated the effects of ruminal microorganisms and metabolites using the 16S rRNA gene sequencing and liquid chromatograph–mass spectrometer (LC-MS) technologies. We found that compared to traditional grazing feeding, house feeding significantly improved the growth performance (such as average daily gain and net meat weight) and rumen development of the yaks. At the genus level, the abundance of *Rikenellaceae RC9 Gut group*, *Christensenellaceae R-7 group*, *Lachnospiraceae NK3A20 group*, *Ruminococcaceae UCG-014*, and *Prevotellaceae UCG-003* showed significant differences and was closely related to rumen development in the two distinct feeding systems. Also, metabolomics revealed that the change in the feeding system significantly affected the concentration and metabolic pathways of the related rumen metabolites. The metabolites with significant differences were significantly enriched in purine metabolism (xanthine, adenine, inosine, etc.), tyrosine metabolism (L-tyrosine, dopaquinone, etc.), phenylalanine metabolism (dihydro-3-caumaric acid, hippuric acid, etc.), and cAMP signaling pathway [acetylcholine, (-)-epinephrine, etc.]. This study scientifically support the house fattening feeding system for yaks. Also, our results provide new insights into the composition and function of microbial communities that promote ruminal development and in general growth of the yaks.

## Introduction

The yak (*Bos grunniens*), an outstanding livestock of Qinghai-Tibet Plateau and adjacent areas, provides basic materials of living to the people on the plateau. For several centuries, yak has been an integral part of plateau history and culture, making a great contribution to the Asian plateau economy (Liang et al., 2016). In past years, though the management and breeding techniques have been improved, the traditional concept of grazing is still popular in local herdsmen. It forces yaks to adapt to the local environment of natural pasture mostly with a single food source (Long et al., 1999). Meanwhile, overgrazing has led to serious grassland degradation in the Qinghai-Tibet Plateau (Wang and Fu, 2004; Klein et al., 2007; Miao et al., 2015). Therefore, to alleviate this situation, it has become vital to change the concept of grazing feeding system to house feeding. Moreover, nutritional management, including the change in the traditional feeding system, can improve the overall health and growth performance of yaks.

In ruminants, rumen functions as an important intermediate between light energy absorbed through photosynthesis and the production of easily digestible compounds such as milk and meat (Jami et al., 2014). Rumen, involving complex rumen microbial communities including bacteria, archaea, fungi, and protozoa, can transform indigestible plants into nutrients and energy (Flint et al., 2008). In the rumen microbial community, bacteria account for about 95% and are the most active microorganisms (Brulc et al., 2009; Deusch et al., 2017). Notably, ruminal motility is closely related to microbial fermentation in the rumen that ensures normal physiological activities. Previous studies showed that rumen community composition is extremely sensitive to foods or feeding patterns (Henderson et al., 2015; Zhang J. et al., 2017). For instance, high-concentrate diets improve the proliferation and differentiation of rumen epithelial cells and the expression of transforming growth factor-β1 (TGFB1) and the transcription factor PPAR-α to promote the development of rumen epithelial papilla (Connor et al., 2014). Besides, rumen microbial metabolites facilitate the self-proliferation of microorganisms and also interact with other metabolic factors to control microbial metabolism and related nutritional pathways (Saleem et al., 2013; Bannink et al., 2016). However, so far, there is limited knowledge of microbial composition and rumen metabolites concerning yak rumen development and change in the feeding systems. A comprehensive analysis of this kind can provide an important insight into the microbial metabolic process that may aid animal husbandry production, including yak.

We hypothesized that the change in feeding regimes (grazing → house fattening) could affect the yak rumen microbiota and metabolites, thereby influencing the development of the rumen and the growth performance of the yak. Surprisingly, there is no comprehensive study of analyzing the effects of different feeding systems in yaks so far. Accordingly, here, we comprehensively analyzed the effects of two different feeding systems on the growth of rumen, ruminal microorganisms, and their metabolites in yaks. For this, we combined 16s rRNA sequencing technology with LC-MS to study the community of yak rumen bacteria and discussed the possible relationship between rumen microorganisms and metabolites, which possibly influences the rumen development and individual growth of the animal.

## Materials and Methods

### Animal Welfare

Lanzhou Institute of Husbandry and Pharmaceutical Sciences of the Chinese Academy of Agricultural Sciences (CAAS) approved all animal experiments, and the grant number is 1610322020018. All the slaughter and sampling procedures strictly complied with the Guidelines on Ethical Treatment of Experimental Animals of China.

### Animals, Feeding Regimes, and Weight Determination

Twenty healthy male yaks were selected from Datong County, Qinghai Province, China, and were randomly divided (10 in group each) into grazing (Group G) and stabling group (Group HF). This study started in May and lasted for 160 days, with the first 10 days as preadaptation. All experimental animals were dewormed before the test and weighed every 30 days before morning feeding or grazing. Group HF was fed on total mixed ration (TMR) that were compounded according to the total energy required for daily gain of 400 g for 200 kg beef cattle (shown in Supplementary Table 1). Group G (control group) was grazed in the natural grassland without supplementary feed.

### Sample Collection and Measurements

All yaks were fasted for 24 h and water-deprived for 8 h after the end of the test period. Then, six yaks were randomly selected from each group to collect the rumen fluid using a flexible oral stomach tube with a metal strainer. The tools were washed with clean warm water during collection of rumen fluid, and the first 100 ml of fluid was discarded to eliminate saliva contamination. In the end, the 50-ml rumen fluid of each yak was collected to be measured for rumen pH by pH meter (PHBJ-261L, INESA, Shanghai, China). The samples were divided into 10-ml sterile centrifuge tubes and stored in liquid nitrogen for further tests.

Subsequently, 12 yaks were slaughtered to measure carcass weight and net meat weight following the methods of “Cattle Production Science” (Mo, 2010). Also, rumen tissues were collected for histological observation. For that, the tissues were fixed in 4% paraformaldehyde, and then the steps of dehydration, pruning, embedding, slicing, dyeing, and sealing were carried out in sequential order. Finally, the rumen tissues were observed by a digital trinocular camera microscope (BA410Digital, Motic, Xiamen, China), and five indicators, including the length and width of rumen papillae, the thickness of rumen epithelial, stratum corneum, and muscular thickness, were measured by Motic Images Advanced software (version 3.2).

### 16s rRNA Gene Amplification and MiSeq Sequencing

Based on the manufacturer’s instructions, the microbial DNA was extracted by OMEGA Soil DNA Kit. Then, the purity and concentration of DNA was verified by NanoDrop 2000c (ThermoFisher Scientific Inc., Waltham, MA, United States) and 1% agarose gel electrophoresis. V3–V4 variable regions of 16s rRNA genes were PCR (Polymerase Chain Reaction) amplified with primers 343F (5′-TACGGRAGGCAGCAG-3′) and 798R (5′-AGGGTATCTAATCCT-3′) via ABI GeneAmp^®^ 9700 (ABI, United States) with TransStart^®^ Fastpfu DNA Polymerase (TransGen, Shanghai, China). The total PCR reaction volume was 50 μl containing 10 μl of 5 × TransStart^§^ FastPfu Buffer, 4 μl of dNTPs (2.5 mM), each 1 μl of Forward Primer (10 μM) and Reverse Primer (10 μM), and 10 ng template DNA; the rest was added with autoclaved distilled water to 50 μl. The amplified PCR products were analyzed by 2% agarose gel electrophoresis, and AxyPrep DNA Gel Extraction Kit (Axygen Biosciences, Union City, CA, United States) was used for DNA purification. The final purified products were quantified by QuantiFluor dsDNA System (Promega, United States) following the manufacturer’s instruction.

Equal amounts of purified amplicon were pooled to construct the paired-end sequencing libraries, which were sequenced using the Illumina MiSeq platform (Illumina, San Diego, CA, United States) by Majorbio Bio-Pharm Technology Co., Ltd. (Shanghai, China) following the standard protocols.

### Sequence and Rumen Microflora Processing

The raw sequencing data in FASTQ files were processed and quality-filtered using Trimmomatic software (Bolger et al., 2014). Paired-end reads were assembled using the FLASH software (Reyon et al., 2012) with the following assembly parameters: 10 bp of minimal overlapping, 200 bp of maximum overlapping, and 20% of maximum mismatch rate. Then, the ambiguous, homologous, or <200 bp reads were excluded, and the reads with 75% of bases above Q20 were retained. Also, the chimeric reads were removed. The last two steps were performed using the QIIME software (version 1.9.1^1^) (Caporaso et al., 2010). After removal of primer sequences, clean reads were clustered to generate the operational taxonomic units (OTUs) using the UPARSE software (version 7.0.1090^2^) with 97% similarity cut-off (Edgar, 2013). The representative read of each OTU was selected using QIIME package (version 1.9.1^1^). All representative reads were annotated and blasted against Silva 16s rRNA database (version 132^3^) using RDP classifier algorithm (version 2.11^4^) with 70% of confidence threshold (Wang et al., 2007).

Alpha diversity indexes were performed by MOTHUR (version v1.30.2) (Schloss et al., 2009). The ACE estimator (ACE) and Chao1 Richness Index (Chao1) were used to analyze the richness of the community, and Shannon indices, Simpson, and Good’s coverage index were used to analyze the community diversity.

Beta diversity was calculated based on the unweighted UniFrac distance, and the results were visualized via principal coordinate analysis (PCoA) and plotted according to GUniFrac and ape packages in R (Chen et al., 2012; Paradis and Schliep, 2019). To distinguish significant differences in the abundance at the phyla and genera levels, we used Statsp package in R and SciPy package in PYTHON along with Wilcoxon rank-sum test within STAMP (Jones et al., 2001; Parks et al., 2014).

### LC-MS Metabolomics Analysis of Rumen Fluid

All 12 rumen fluid sample tubes were thawed at room temperature (RT), and then 100 μl of each sample was transferred into a new 1.5-ml centrifuge tube. The samples were vortexed for 30 s after the addition of 300 μl of methanol and 10 μl of internal standard (3.0 mg/ml, DL-o-chlorophenylalanine). The mixture was then centrifuged (12,000 rpm/min, 4°C) for 15 min, and the respective supernatants were transferred to a fresh vial for LC-MS (Thermo, Ultimate 3000LC, Q Exactive). A preheated hyper gold C18 column (100 × 4.6 mm, 3 μm internal diameter) was used for chromatographic separation in positive ion mode (ESI +) and negative ion modes (ESI-). The samples were eluted with a mobile phase consisting of solvent A (water and 5% acetonitrile with 0.1% formic acid) and solvent B (acetonitrile with 0.1% formic acid) with a flow rate of 0.35 ml/min. The elution was performed in three steps, with mobile phase proportions (A:B) 100%:0% for 1 min, 80%:20% for 1.5 min, and 0%:100% for 9.5 min, respectively.

Other relative conditions were as follows: Sheath Gas 45 arb, Aux Gas 15 arb, Sweep Gas 1 arb, ion source temperature: 300°C and capillary temperature: 350°C. Also, the quality control (QC) samples consisting of equivalent mixtures of all rumen fluids samples were analyzed regularly to ensure the reliability of data.

### Metabolomics Data Analysis

Baseline filtration, peak identification, peak alignment, peak filling, and retention time (RT) of the raw data were performed by Progenesis QI software (Waters Corporation, Milford, United States). Finally, a data matrix of RT, mass–charge ratio (MZ), and peak strength was obtained. To observe the metabolic changes between groups, principal component analysis (PCA) and orthogonal partial least squares discriminant analysis (OPLS-DA) were performed by the R package ropls (Version 1.6.2), and 7-fold cross validation was used to evaluate the model stability. The significantly different metabolites were selected based on the combination of the variable important in projection (VIP) obtained from the OPLS-DA model and the Student’s *t*-test. The metabolites with VIP > 1 and *P* < 0.05 were considered as significantly different metabolites.

For hierarchical clustering of each sample, we used the qualitative data of significantly different metabolites in the gplots package in R (Warnes et al., 2005) to accurately screen marker metabolites and study the alterations in related metabolic processes. For Spearman correlation analysis, we used the pheatmap package in R (Kolde, 2012), and *P*-values < 0.05 were selected as statistically significant.

The dataset for 16s rRNA gene sequencing and metabolome was deposited in 10.6084/m9.figshare.14538567.v1.

## Results

### Characterization of Growth Performance, Rumen pH, and Morphological Development of Yak

The characteristics of yak growth performance are listed in Table 1. The total average body weight gain and average daily weight gain in group HF were 161.50 kg and 860 g, respectively. On the contrary, in group G, the total average body weight gain and average daily weight gain were 98.50 kg and 520 g, respectively. Also, we observed a significant difference (*P* < 0.01) in carcass weight and net meat weight between the two groups.

**TABLE 1:** The growth performance of yaks in different feeding systems.

Items	Group (mean ± SD)		*P*-value
	G (kg)	HF (kg)	
Initial body weight	208.33 ± 12.53	215.67 ± 21.83	0.6549
Final body weight	306.83 ± 24.34 ^*B*^	377.17 ± 15.77 ^*A*^	0.0010
Average daily gain	0.52 ± 0.12 ^*b*^	0.86 ± 0.15 ^*a*^	0.0017
Carcass weight	124.28 ± 8.77 ^*B*^	208.04 ± 8.75 ^*A*^	< 0.001
Net meat weight	94.73 ± 4.55 ^*B*^	176.23 ± 10.61 ^*A*^	< 0.001

*Different small and capital letters represent the significant (*P* < 0.01) and highly significant difference (*P* < 0.001), respectively. SE: Standard Error.*

**FIGURE 1:**
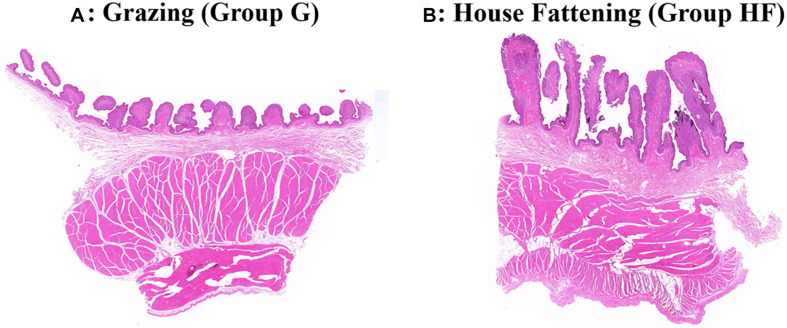
Effects of different feeding systems on the morphological development of yak rumen. **(A)** The grazing group. **(B)** The house fattening group.

The microstructure of the rumen epithelium is shown in Figure 1. Besides, Table 2 presented the six measured indicators of rumen. We observed that the length and width of rumen papillae (*P* < 0.01), the thickness of rumen epithelial, and the stratum corneum (*P* < 0.05) were significantly lower in group G than in group HF. Meanwhile, the rumen pH in group G was significantly higher due to the change in feeding regimes (*P* < 0.05).

**TABLE 2:** Effects of different feeding systems on various indicators of rumen.

Items	Group (mean ± SD)		*P*-value
	G	HF	
pH	7.68 ± 0.31^*a*^	7.21 ± 0.25^*b*^	0.014
Papilla length (μm)	1529.03 ± 157.87^*B*^	2204.39 ± 239.95^*A*^	0.0031
Papilla width (μm)	346.91 ± 48.65^*B*^	444.88 ± 25.67^*A*^	0.0014
Epithelial (μm)	70.23 ± 10.52^*b*^	90.43 ± 12.71^*a*^	0.021
Stratum corneum (μm)	20.32 ± 1.93^*b*^	23.46 ± 2.79^*a*^	0.046
Muscle layer (μm)	3681.24 ± 440.22	3106.13 ± 367.51	0.445

*Different small and capital letters represent the significant (*P* < 0.05) and highly significant difference (*P* < 0.01). SE: Standard Error.*

### Richness, Diversity Estimates, and Rumen Bacteria Composition

In total, from all of the rumen liquid samples, we retained 587,595 sequences as the high-quality dataset. After extracting non-repeating sequences from optimized sequences and excluding the non-repeating single sequences, a total of 2,619 OTUs were obtained by clustering at 97% identity. The sampling depth, signifying the optimal evaluation of rumen bacterial composition, is reflected by the Shannon index and Sobs index curves. Supplementary Figure 1 shows that curves gradually stabilized after initial rising, indicating the depth of sample sequencing that covered most of the intestinal flora, which was used for subsequent data analysis. Besides, to access the sufficient OTU coverage of samples, we found that the Good’s coverages of all the samples exceeded 99%, suggesting the accuracy of sequencing data that covered all the species in the sample.

**FIGURE 2:**
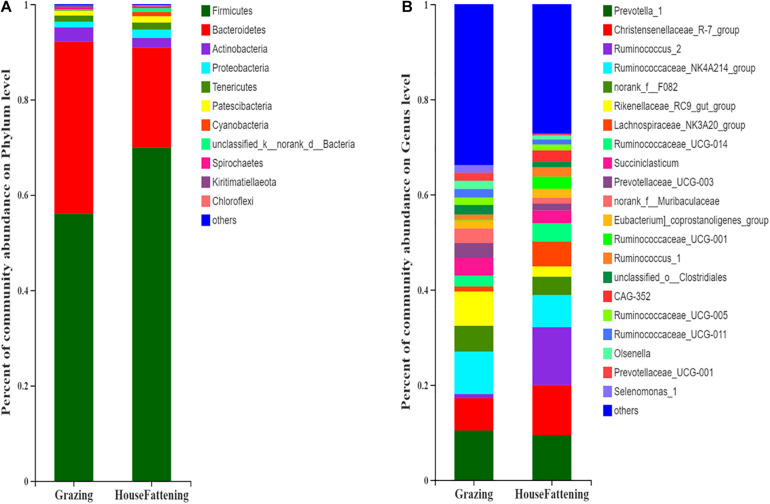
Bacterial community compositions across the two feeding systems. **(A)** Phylum level; **(B)** Genus level.

The Chao value went from 1724.50 ± 116.30 to 1184.30 ± 149.23 (*P* < 0.001), the Shannon index went from 5.925 ± 0.094 to 5.023 ± 0.308 (*P* < 0.001), the Ace index went from 1708.70 ± 118.65 to 1178.20 ± 148.85 (*P* < 0.001), and the Simpson index went from 0.008 ± 0.001 to 0.021 ± 0.007 (*P* < 0.01) (Supplementary Figure 2). This indicates that there were significant differences in microbial diversity and richness between the two different feeding systems. Importantly, we found that there was higher diversity in the grazing group than in the house fattening group. Taxonomic analysis revealed a total of 29 bacterial phyla, including 22 bacterial phyla in both groups and 7 phyla only in the HF group. Among them, *Firmicutes* and *Bacteroidetes* were the dominant phylum with 56.03%, 36.11% in group G and 69.90%, 21.00% in group HF, respectively (Figure 2A). A total 428 different genera was detected in the rumen liquids of yaks at the genus level. Among them, 220 genera were identified in both groups, while 42 and 132 genera were unique for group G and group HF, respectively. *Prevotella 1* (10.40%, 3.40%), Christensenellaceae R-7 group (6.79%, 10.56%), Ruminococcus 2 (0.89%, 12.17%), *Ruminococcaceae NK4A214 group* (8.91%, 6.75%), *Rikenellaceae RC9 gut group* (7.18%, 2.15%), and *Lachnospiraceae NK3A20 group* (1.08%, 5.25%) were predominant genera in group G and group HF, respectively (Figure 2B).

### Differences in Bacterial Community Composition Between the Two Feeding Systems

**FIGURE 3:**
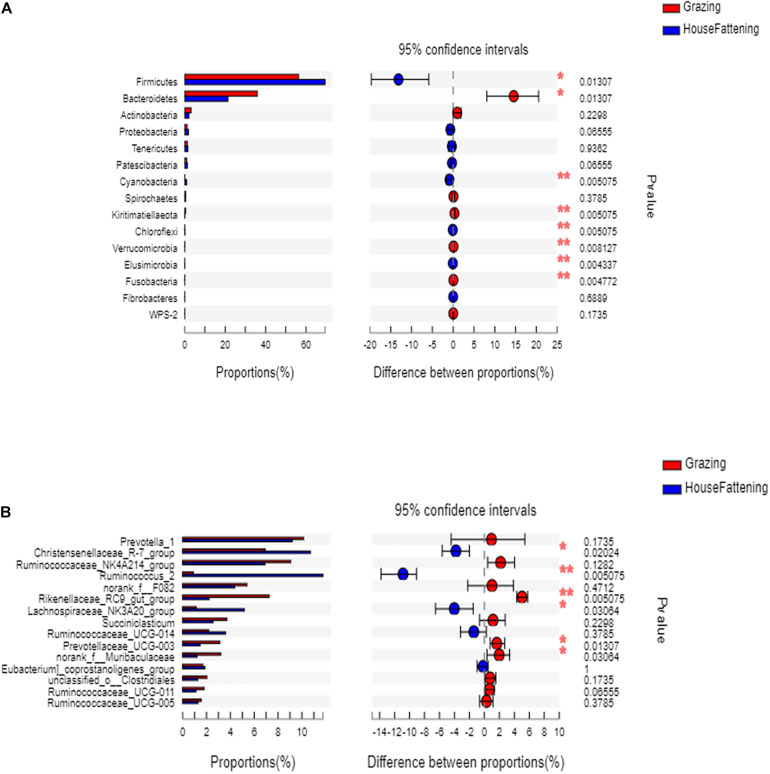
**(A)** The bacteria with significant differences between two feeding systems at the phylum level; **(B)** The bacteria with significant differences between two feeding systems at the genus level. *P < 0.05; **P < 0.01.

At the phylum level, the relative abundances of *Firmicutes*, *Cyanobacteria, chloroflexi*, and *Elusimicrobia* were significantly higher (*P* < 0.05) in the HF group than in the G group (Figure 3A). On the contrary, *Bacteroidetes*, *Kiritimatiellaeota*, *Verrucomicrobia*, and *Fusobacteria* were significantly higher in the G group (*P* < 0.05).

At the genus level, the ruminal microbiome of the grazing group showed a higher abundance of *Rikenellaceae RC9 gut group* and *Prevotellaceae UCG-003* than the stabling group (*P* < 0.05) (Figure 3B). Also, the *Christensenellaceae R-7 group*, *Lachnospiraceae NK3A20 group*, and *Ruminococcaceae UCG-014* in the grazing yak were significantly higher than in stabling yak (*P* < 0.05). Next, to validate the differences in bacterial community between the grazing and house fattening yaks, we used PCoA with unweighted UniFrac matrix distances to reveal the influence of the two feeding systems (Supplementary Figure 3).

### Correlations Between the Indicators of Ruminal Development and Rumen Bacteria

**FIGURE 4:**
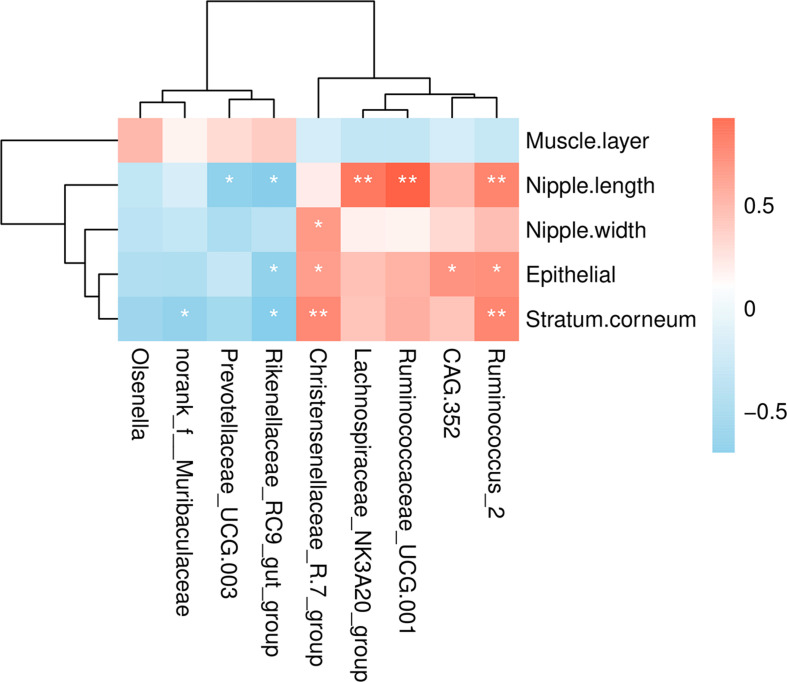
Correlations analysis between ruminal development indicators and rumen bacteria. Rows and columns represent the indicated ruminal development indicator and genus, respectively. Each lattice represents a Pearson correlation coefficient between an indicator with a genus. Red and blue denote positive and negative correlation, respectively. *P < 0.05, **P < 0.01.

To understand the relationship between microbial populations showing significant differences in abundance at the genera level and ruminal development indicators, we constructed a correlation heat map based on Spearman’s correlation coefficient (Figure 4). We found that the *Christensenellaceae R-7 group* and *Ruminococcus 2* were significantly positively correlated with the thickness of epithelium and stratum corneum. Also, these were positively correlated with the rumen nipple width and length, respectively. Likewise, *Ruminococcus 2*, *Ruminococcaceae_UCG 001*, and *Lachnospiraceae NK3A20 group* were significantly positively correlated with the rumen nipple length. Besides, the *Rikenellaceae RC9 gut group* was negatively correlated with the nipple length and the thickness of ruminal epithelium and stratum corneum. Similarly, *Prevotellaceae UCG 003* was negatively correlated with the nipple length.

### Metabolomics of Rumen Samples

**FIGURE 5:**
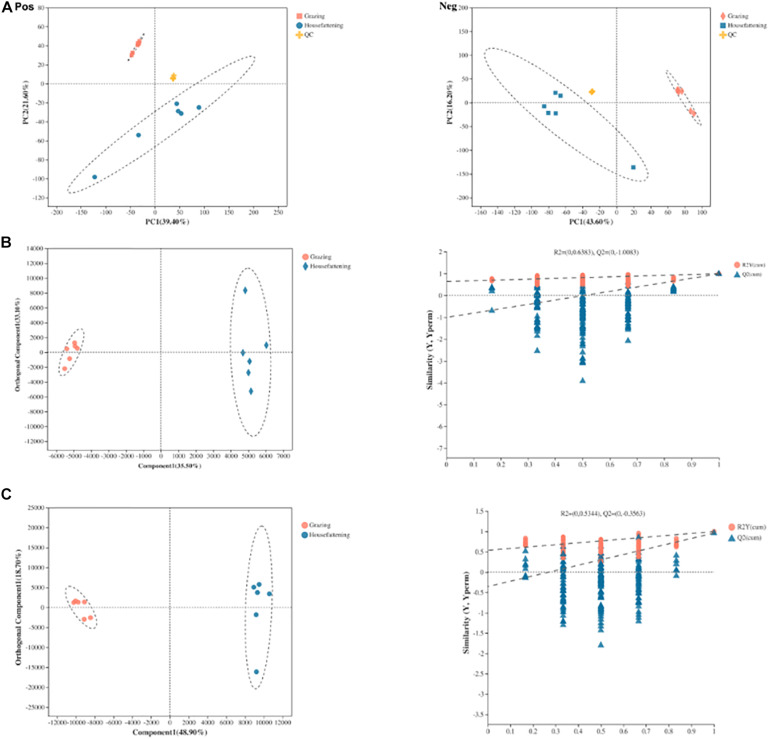
Principal component analysis (PCA) score plots of metabolic profiling in positive (Pos) and negative (Neg) ion mode **(A)**. OPLS-DA models of positive **(B)** and negative **(C)** mode ionization. Different colors represent different groups; pink, grazing group; blue, house fattening group; yellow, QC group.

Ruminal fluid metabolites from grazing and house fattening groups, including QC samples, were analyzed by LC-MS. The PCA following positive and negative mode ionization showed a primary unsupervised separation between the two groups including QC samples (Figure 5A). To better distinguish the differences between the groups and improve effectiveness, OPLS-DA with positive and negative mode ionization was performed to supervise the multivariate analysis (Figures 5B,C). We found that the two groups were separated clearly and all the samples in the score plots were in the 95% Hotelling T2 ellipse, indicating the validity of the OPLS-DA model.

As shown in Supplementary Table 2, there were 177 differential metabolites, including 49 positively and 128 negatively ionized metabolites, between the grazing and house fattening group with a variable importance projection (VIP) value >1.0 and *P* < 0.05. The 49 positively ionized metabolites included 26 lipids and lipid-like molecules, 4 organoheterocyclic compounds, 3 nucleosides, nucleotides, and analogs, 1 organic oxygen compounds, 1 organic nitrogen compounds, 2 benzenoids, 7 organic acids and derivatives, 1 phenylpropanoids and polyketides, 3 alkaloids and derivatives, and 1 organooxygen compounds. Similar to the positively ionized metabolites, the more differential metabolites were divided into more categories, including 76 lipids and lipid-like molecules, 9 organic acids and derivatives, 10 organoheterocyclic compounds, 12 phenylpropanoids and polyketides, 6 nucleosides, nucleotides, and analogs, 6 benzenoids, 6 organic oxygen compounds, and 3 lignans, neolignans, and related compounds.

**FIGURE 6:**
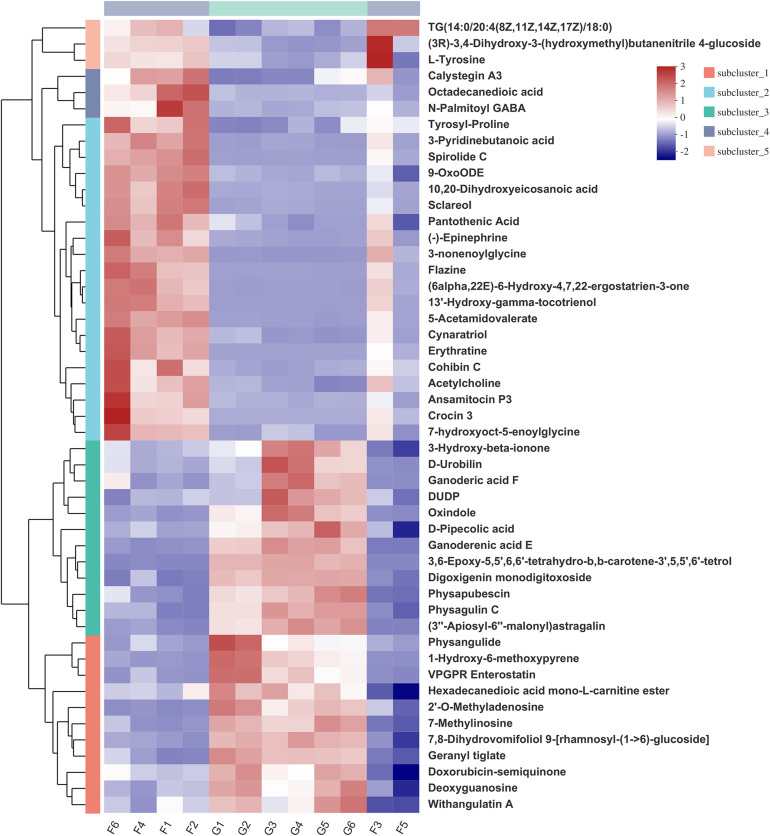
Hierarchical clustering analysis (HCA) with a heat map for the expression of different metabolites in yak rumen between grazing group and house fattening group following positive mode ionization.

**FIGURE 7:**
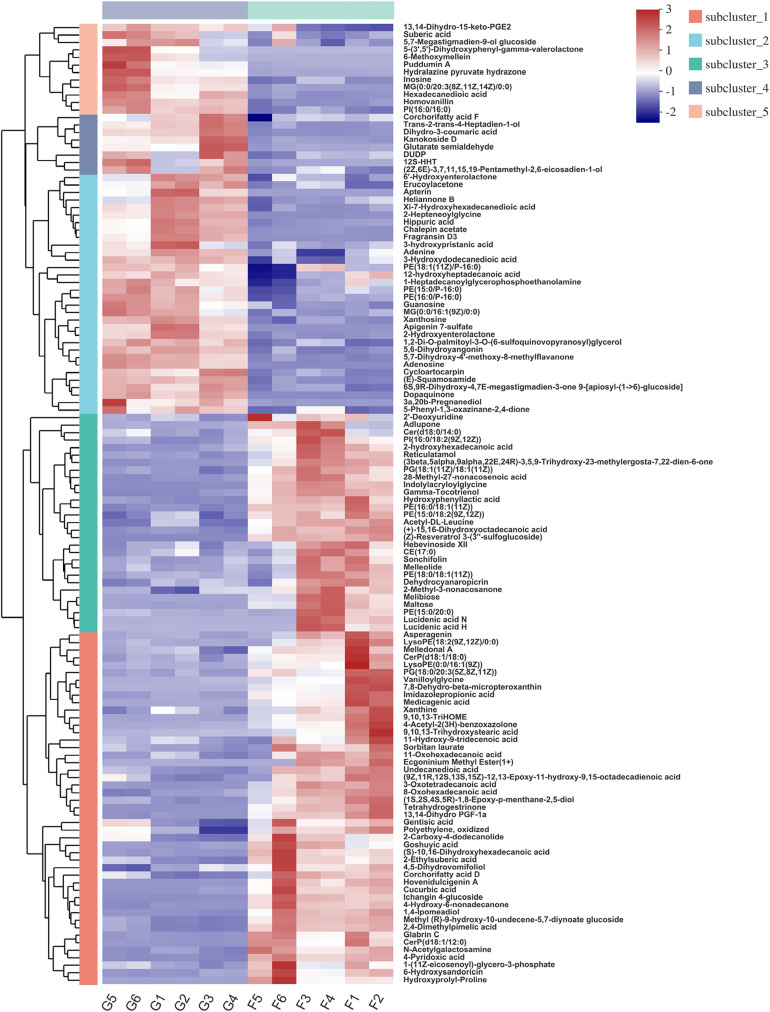
Hierarchical clustering analysis (HCA) with a heat map for the expression of different metabolites in yak rumen between grazing group and house fattening group following negative mode ionization.

Hierarchical clustering analysis (HCA) with a heat map allowed the visualization of the expression of concentrated metabolites in each sample. Besides, it distinctly revealed the metabolome differences between the two feeding systems. HCA heat maps for the positive and the negative ionization data are shown in Figures 6, 7. The differential metabolites in the positive mode ionization group were divided into five distinct clusters. Cluster 1 consisted of 11 metabolites, such as 7-methylinosine, hexadecanedioic acid mono-L-carnitine ester, and deoxyguanosine. Cluster 2 included pantothenic acid, acetylcholine, and 18 other metabolites. Cluster 3 contained 12 metabolites, including D-Urobilin, DUDP, D-Pipecolic acid, and 9 other metabolites. Cluster 4 contained octadecanedioic acid, calystegin A3, and N-Palmitoyl GABA. Cluster 5 included L-Tyrosine and two other metabolites. Similarly, in the negative ionization group of compounds, Cluster 1 included gentisic acid, xanthine, cucurbic acid, and 44 other metabolites. Cluster 2 consisted of adenine, hippuric acid, adenosine, and 29 other metabolites. Cluster 3 had 29 metabolites such as hydroxyphenyllactic acid, acetyl-DL-leucine, and gamma-tocotrienol. Cluster 4 contained dihydro-3-coumaric acid, glutarate semialdehyde, and six other metabolites. Cluster 5 included inosine, suberic acid, and hexadecanedioic acid. Hierarchical clustering analysis heatmap revealed a significant effect on the rumen metabolome between the grazing and house fattening yaks. For instance, compared to the G group, in the HF group, among the clusters belonging to the positive ionization group, clusters 2, 4, and 5 were upregulated, whereas clusters 1 and 3 were downregulated. Similarly, among clusters belonging to the negative ionization group, clusters 1 and 3 were upregulated, while clusters 2, 4, and 5 were downregulated.

### KEGG Enrichment Analysis and Correlations Between the Differential Metabolites and Rumen Bacteria

**FIGURE 8:**
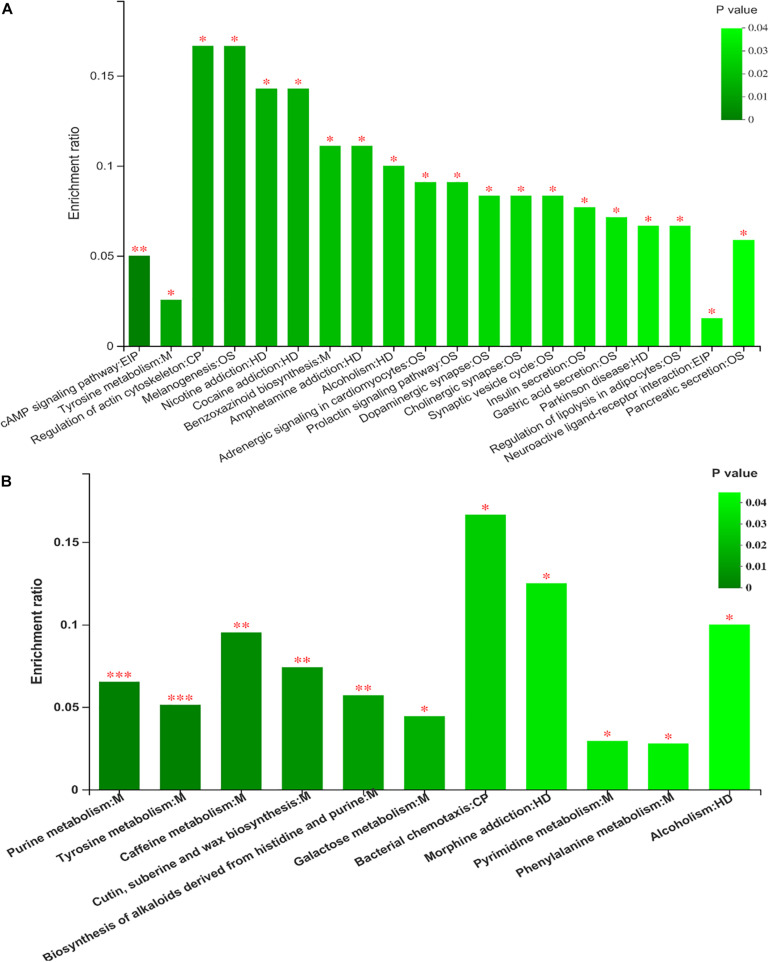
The analysis of different metabolites pathway enrichment **(A)** positive mode ionization; **(B)** negative mode ionization. *P < 0.05, **P < 0.01.

**FIGURE 9:**
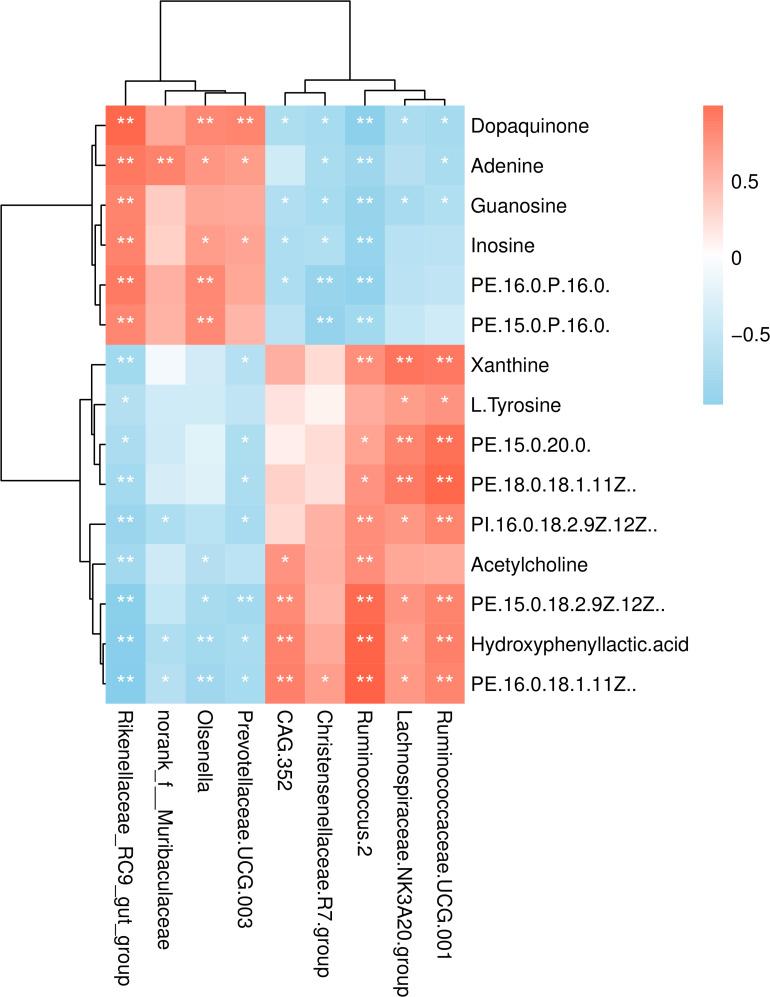
Correlation analysis between the rumen microbiome and differential metabolites. Row, columns, and lattice represent the metabolites, genus, and Pearson correlation coefficient between a component and metabolite, respectively. *P < 0.05, **P < 0.01.

Kyoto Encyclopedia of Genes and Genomes (KEGG) enrichment analysis revealed that the identified differential metabolites between the two feeding systems were mainly enriched in purine, tyrosine, and phenylalanine metabolism, cAMP signaling pathway, and so on (Figure 8). To understand the relationship between the composition and function of microbial communities, we performed a correlation analysis between the rumen microbiome and differential metabolites (Figure 9). We found that the dynamic fluctuations in some metabolites were closely related to the abundance of various microbial communities. Among them, genus *Ruminococcus 2* was negatively associated with dopaquinone, adenine, guanosine, and inosine but was positively associated with xanthine, acetylcholine, and hydroxyphenyllactic acid. Likewise, genus *Ruminococcaceae_UCG 001* was negatively associated with dopaquinone, adenine, and guanosine but was positively associated with xanthine, L-tyrosine, and hydroxyphenyllactic acid. Genus *Lachnospiraceae NK3A20 group* was positively associated with xanthine, hydroxyphenyllactic acid, PE, and PI but was negatively associated with dopaquinone and guanosine. Genus *Christensenellaceae R-7 group* was negatively associated with dopaquinone, adenine, guanosine, inosine, and PE. Genus *Rikenellaceae RC9 gut group* was positively associated with dopaquinone, adenine, guanosine, and inosine but was negatively associated with xanthine, L-tyrosine, acetylcholine, and hydroxyphenyllactic acid.

## Discussion

The yak lives in an extremely special environment usually with a long period of withered grass every year. However, in past years, under the traditional grazing system, the growth performance of yaks has been reduced significantly. This has markedly limited the economic benefits forcing a significant increase in livestock causing overgrazing and grassland degradation in the plateau region (Shang et al., 2014). In this study, we found that compared to the grazing yaks, the average daily weight gain and average net meat weight of group HF were increased 0.34 kg and 81.50 kg, respectively, suggesting that house feeding can increase the growth performance, thereby exerting the genetic potential of the yak’s excellent traits for better economic benefits.

In ruminants, the rumen is like a large anaerobic fermentation tank, in which rumen microorganisms ferment and degrade plants, or convert non-digestible plant feeds into volatile fatty acids (VFA). Here, to comprehensively analyze the effects of different feeding systems on the yak rumen, we analyzed the morphological structure of the rumen and evaluated rumen development indicators. The rumen nipple length is the most important factor that can reflect the impact of different feeding treatments on rumen development (Lesmeister et al., 2004). Also, the rumen epithelium is very important for the absorption of the final fermentation product, as 50 to 85% of VFA are absorbed directly through the rumen epithelium, and the absorption rate of VFA by the ruminal epithelium is highly dependent on the papillary surface area and the availability of transport proteins (Bannink et al., 2008; Melo et al., 2013). We found that the rumen nipple length, width, and epithelial thickness in the HF group were significantly better than in the G group. These changes may be due to the lack of VFA productivity in the grazing yaks without the concentrated feed (Nocek and Kesler, 1980). However, the thickness of the rumen muscular layer became lower in group HF. It may be a reason that the food of the grazing yaks was all pasture, as a kind of roughage that stimulated the muscle development of the rumen wall and thicken the muscle layer. Concisely, these evidences indicate that the change in feeding regime would accordingly alter the rumen morphology. For instance, the HF regime improved the development of rumen papilla for better absorption of VFA. Previous studies showed that the nutrient absorption and transport by the ruminal epithelium largely depends on the degree of keratinization of stratum corneum cells and the integrity of the stratum corneum (Baldwin and Jesse, 1992). In our study, the ruminal stratum corneum of yaks in group HF was significantly higher than that of yaks in group G, indicating increased nutrient absorption by promoting the growth and development of the HF yaks.

Furthermore, we explored the impact of feeding regimes on the composition and diversity of the rumen bacterial community. Previous studies suggest that the core rumen microbiota consists of 10 different bacterial groups in ruminants (such as beef cattle), although the relative abundance can vary (Petri et al., 2013). Consistent with previous studies (Leng et al., 2011; Dan et al., 2016; Zhou et al., 2017; Liu et al., 2019), here, we found that the phyla *Bacteroidetes*, *Firmicutes*, and *Proteobacteria* form the rumen core microbiome in the yaks. Also, the high relative abundance of the phyla *Acidobacteria* indicated that it was the highest conserved dominant microbial community in the rumen. At the genus level, *Prevotella 1*, *Christensenellaceae R-7 group*, *Ruminococcus 2*, *Rikenellaceae RC9 gut group*, and *Ruminococcaceae NK4A214 group* showed high abundance, and they were the dominant bacteria in rumen of yaks. Moreover, *Lachnospiraceae NK3A20 group*, *Ruminococcaceae UCG-014*, *Prevotellaceae UCG-003*, and *Succiniclasticum* were also identified. However, a previous study shows that none of the clones were more than 97% similar to the known genus *Prevotella* with 16s rRNA sequence cloning technology in two different feeding systems. We speculated that it may be due to the difference in the sequencing method and the technical deviations in 16S rRNA gene PCR (Fang et al., 2015). According to the indicators, the rumen bacterial diversity and richness of the grazing yaks were significantly higher than the house fattening yaks. This was contrary to previous research that showed that the rumen microbial diversity of the grazing yaks was lower than that of the house fattening yaks (Cao et al., 2016). We speculate that this may be due to the small sample size in the previous study and the difference in yak breed. Overall, we found that the yak rumen microbial composition was directly related to the different feeding systems.

The two feeding systems revealed significant differences showing that *Christensenellaceae R-7 group* and *Ruminococcus 2* were highly abundant in grazing yaks than in the house fattening yaks. Notably, *Firmicutes* and *Christensenellaceae* have been closely linked to animal health, and their relative abundance was shown to affect the body mass index (Goodrich et al., 2014). Previous studies reported that *Christensenellaceae* could quickly respond to an increase in animal or plant products in the diet (David et al., 2014). Moreover, *Christensenellaceae* is positively correlated with protein catabolism and intestinal metabolites of dietary animal protein (Roager et al., 2016; Beaumont et al., 2017; Manor et al., 2018). Similarly, the change in the abundance of *Ruminococcus*, once considered to be the main fiber-degrading bacteria in the rumen, can significantly alter the digestion and utilization of rumen nutrients (Doerner and White, 1990; Purushe et al., 2010). Also, *Ruminococcus* growth has been be positively correlated with the expression of Toll-like receptor (TLR) genes. These genes can recognize the host’s outer wall bacteria and the induction of bacterial products, and trigger an immune response to maintain host–microbe homeostasis (Liu et al., 2015). Similarly, a previous study regarding 16s rRNA gene libraries indicated that *Ruminococcus* was found in grazing and house feeding systems and the content was higher in grazing yak rumen (Fang et al., 2015). These results show that genus *Ruminococcus* may act as an important part in yak rumen. In our study, we found that *Christensenellaceae R-7 group* was significantly positively correlated with nipple width, epithelial thickness, and stratum corneum thickness. *Ruminococcus 2* was significantly positively correlated with nipple length, epithelial thickness, and stratum corneum thickness. These results indicate that with an increase in abundance of *Christensenellaceae R-7 group* and *Ruminococcus 2*, the rumen development improves and the rumen becomes more conducive to the absorption and digestion of nutrients. Similarly, the abundance of *Lachnospiraceae NK3A20 group* was significantly increased in the house fattening yaks, and it was positively correlated with rumen nipple length. *Lachnospiraceae*, the main component of the gastrointestinal microbiota of ruminants (Kittelmann et al., 2013), is closely related to the butyrate production (Vital et al., 2014; Haas and Blanchard, 2017). It was reported that the infusion of butyric acid could increase the length, width, and surface area of the rumen epithelial papilla of castrated cattles (Xu et al., 2001). Here, we speculate that *Lachnospiraceae NK3A20 group* not only promotes the rumen development by promoting butyric acid production but also affects the rumen development directly. By increasing the abundance of *Lachnospiraceae NK3A20 group*, the efficiency of the yak’s rumen was improved so that the yak could get more energy and accelerate the growth and development. Also, we found that the abundance of *Rikenellaceae RC9 gut group* in group HF was significantly lower and was negatively correlated with the length of the ruminal papilla, epithelial thickness, and stratum corneum thickness. *Rikenellaceae RC9 gut group*, belonging to the *Rikenellaceae* family, plays an important role in the digestion of crude fiber (Zhang, 2017). Previous studies showed that when the content of neutral detergent fiber in the diet is reduced from 39.7 to 30.9%, the relative abundance of *Rikenellaceae RC9 gut group* in the rumen decreased by 69.8% (Zened et al., 2013). Therefore, the decrease of roughage content in the house fattening group in our study could explain the decrease of *Rikenellaceae RC9 gut group* in the rumen of the house fattening yak. *Prevotellaceae UCG-003* was significantly reduced in the HF group and negatively correlated with the length of the rumen papilla. *Prevotellaceae UCG-003*, classified under the *Prevotellaceae* family, is sensitive to rumen pH (Mao et al., 2016). It seems that a decrease in rumen pH of the house fattening yak reduced the abundance of *Prevotellaceae UCG-003*. Previously, it was found that the predominant *Prevotella* can improve feed utilization (Purushe et al., 2010), and *Prevotellaceae UCG-003* can utilize branched-chain VFAs and participate in glucose metabolism (Liu et al., 2019). This explains the decrease of *Prevotellaceae UCG-003* abundance in the HF group; however, the mechanism of reduced *Prevotellaceae UCG-003* induced nipple length needs further exploration.

Metabolomics can better explain the change in phenotypes than genomics and proteomics (Vinayavekhin et al., 2010). Our metabolomics data showed that the alteration in feeding mode did also change the concentration of many rumen metabolites, which could be related to the variation of rumen microbial abundance. Next, we explored the key metabolic pathways based on impact values and *P*-values (Chen et al., 2018; Wang et al., 2018; Yang et al., 2018). The metabolites with significant difference that were screened were mainly enriched in tyrosine metabolism, purine metabolism, phenylalanine metabolism, and cAMP signaling pathway. Overall, the relative concentrations of carbohydrates, amino acids, purines, and other metabolites in the rumen of house fattening yaks were significantly higher than the grazing yaks. Amino acids are important for the growth and metabolism of microorganisms. They are also the key components in the synthesis of proteins and peptides and regulate several metabolic pathways (Mariz et al., 2018). Tyrosine is an essential aromatic amino acid, and both phenylalanine and tyrosine metabolism were related to tyrosine, indicating that tyrosine may play an important role in the change of feeding system. Previous studies showed that tyrosine metabolism is instrumental in synthesis of thyroid hormones, catecholamines, and melanin (Zhang et al., 2004). Also, the rumen tyrosine content in fattening sheep was found positively correlated with the production performance (Li, 2020). Here, tyrosine was significantly upregulated in the HF group, which can be converted to tyrosamine through decarboxylation reaction (Burlingame and Chapman, 1983). Notably, excessive accumulation of tyramine, a biogenic amine, can prevent epithelial cell regeneration, leading to epithelial damage in the rumen (Mao et al., 2016). Fortunately, we found that the alteration of feeding system did not significantly increase the tyramine content in the rumen. Meanwhile, we found that the concentration of dopaquinone, involved in tyrosine metabolism, was significantly reduced in group HF. Dopaquinone is formed from dopamine by tyrosinase, and an increase in dopamine affects the synthesis and secretion of growth hormone (GH) to promote the growth of animals (Terry and Craig, 1985). A significant decrease in dopaquinone in group HF may increase dopamine, thereby promoting the growth and development of house fattening yaks. (-)-Epinephrine was increased significantly in the rumen of house fattening yaks; however, its effects on rumen remain to be clarified. Carbohydrate, such as maltose and melibiose, were significantly increased in the HF group. These disaccharides can be hydrolyzed to produce glucose for growth and development of body. Starch, as a non-fiber carbohydrate, can be rapidly degraded into maltose, maltotriose, and a small amount of free glucose by the breakage of α-D-1,4-glycosidic bond by α-amylase produced from the amylolytic bacteria of the rumen (Reynolds, 2006). It was reported that some *Prevotella* genera can degrade nutrients such as protein, starch, and xylan (Purushe et al., 2010). This explains the improved ability of HF yak to degrade nutrients with an increase in *Prevotella* abundance. At the same time, rumen microorganisms also need to maintain their growth by producing lactic acid to further produce propionic acid using molasses and maltose (Zhu and Mao, 2011). An increase of propionic acid can improve the internal environment and promote the growth and development of the rumen, protein synthesis, and digestive enzyme activity, which is conducive to the absorption of nutrients and improves animal growth performance.

Notably, we found that the concentrations of several metabolites enriched in the purine metabolism were also changed significantly. Usually, the amount of nucleic acid entering the rumen is very low as it is fully degraded in there. Also, the microbial communities in rumen epithelium can degrade epithelial debris, including nucleic acids and their derivatives (Chen et al., 1990). The protozoan in the rumen cannot synthesize purines and pyrimidines but can absorb free adenine and guanine to synthesize nucleic acids for their needs. The nucleic acid of the protozoan in the rumen may mainly come from rumen bacteria (McAllan, 1982). Xanthine is considered as a biomarker for microbial protein synthesis (McAllan and Smith, 1973; Zhang R. et al., 2017). We found that xanthine was significantly increased in HF yaks. Importantly, adenine and guanine can, respectively, produce hypoxanthine and xanthine deamination (Chen et al., 1990), and inosine can also be enzymatically degraded to xanthine (Stentoft et al., 2015). We speculate that change in the feeding system decreased guanine, inosine, and adenine but increased xanthine in the HF group. These results also showed that the transformation from grazing mode to house fattening mode improved purine metabolism in the rumen.

Besides, in the HF yaks, we found significant changes in the concentrations of phosphatidylethanolamines (PE), phosphatidylinositols (PI), and phosphatidylglycerols (PG), which are related to lipid metabolism. Interestingly, the change in PE concentration is consistent with the findings in dairy cows (Li et al., 2012).

Phosphatidylethanolamines (PE) mainly exists in the outer layer of the bacteria cell wall and is a precursor of ethanolamine (Stentoft et al., 2015). It accounts for >75% of total lipids in *Bacteroides*. Notably, the change in the feeding system decreased the rumen pH in the HF group, which could cease the growth and reproduction of *Bacteroides* releasing lipopolysaccharide (LPS) in the rumen (Bannerman et al., 2003; Klevenhusen et al., 2013). However, we did not find significant changes of LPS in the results of the metabolome; therefore, we speculated that the increase of PE in the HF group might be due to the comprehensive effects of the alteration in feeding system that may have promoted the regeneration and shedding of rumen epithelial cells. Phosphatidylinositol transfer protein (PITP) promotes the transport of phospholipid molecules, including PI. Also, it is a precursor for the synthesis of a secondary messenger named 1,4,5-inositol triphosphate. Notably, the PI metabolic process can also be affected by the signaling pathways involving phospholipase C, phosphatidylinositol 3-kinase, membrane transport, and other biological functions, such as glycometabolism, lipid metabolism, and protein metabolism (Jiang and Li, 2016). But up to now, the exact mechanism is unknown and needs to be investigated further.

## Conclusion

We combined the data of growth performance, rumen development, microbiome, and metabolomics to comprehensively analyze the effect of feeding system transformation in yaks. We explored the possible relationship among specific rumen bacterial communities, metabolites, and rumen development in yak. Notably, the metabolomics findings were consistent with animal phenotypes, while the house fattening system provided more nutrients and was conducive to the growth and development of yaks. We show that barn feeding can promote the growth performance and ruminal development of yaks. Comprehensive analysis of the feeding systems effects in yaks can not only improve the economic benefits in Qinghai-Tibet Plateau but also provide a basis for better understanding of metabolites and microbial functions of yaks.

## Data Availability Statement

The original contributions presented in the study are included in the article/Supplementary Material, further inquiries can be directed to the corresponding authors.

## Ethics Statement

The animal study was reviewed and approved by the Lanzhou Institute of Husbandry and Pharmaceutical Sciences of the Chinese Academy of Agricultural Sciences. Written informed consent was obtained from the owners for the participation of their animals in this study.

## Author Contributions

CL, PY, and CH contributed to conception and design of the study. CH, CL, and XY collected the samples. CH, PY, XY, and FG conducted relevant experiments. CH and XY organized the database. FG and XY performed the statistical analysis. CH and FG wrote the first draft of the manuscript. XG, PB, XM, XW, and MC wrote sections of the manuscript. All authors contributed to manuscript revision, read, and approved the submitted version.

## Conflict of Interest

The authors declare that the research was conducted in the absence of any commercial or financial relationships that could be construed as a potential conflict of interest.

## Footnotes

1 http://qiime.org/install/index.html

2 http://drive5.com/uparse/

3 https://www.arb-silva.de/

4 https://sourceforge.net/projects/rdp-classifier
