# Supplementary figures and images for: Microbiome and Metabolomics Reveal the Effects of Different Feeding Systems on the Growth and Ruminal Development of Yaks
Source: Front Microbiol. 2021 Jun 22;12:682989. doi: 10.3389/fmicb.2021.682989 (PMC8265505; doi:10.3389/fmicb.2021.682989)

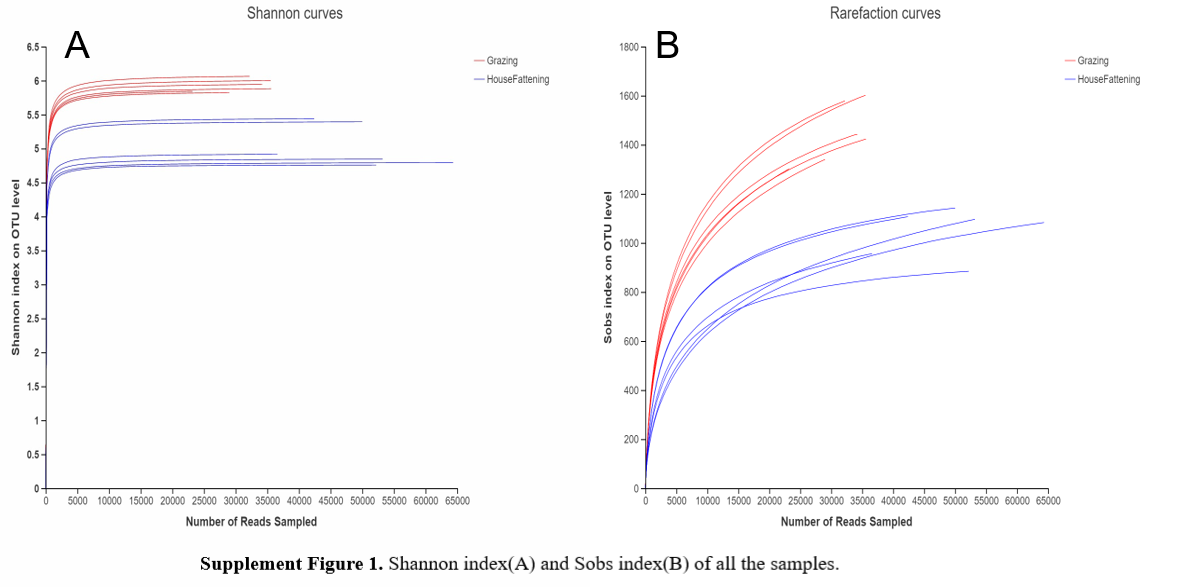

Supplement: Supplementary file 1 [file Image_1.TIF]

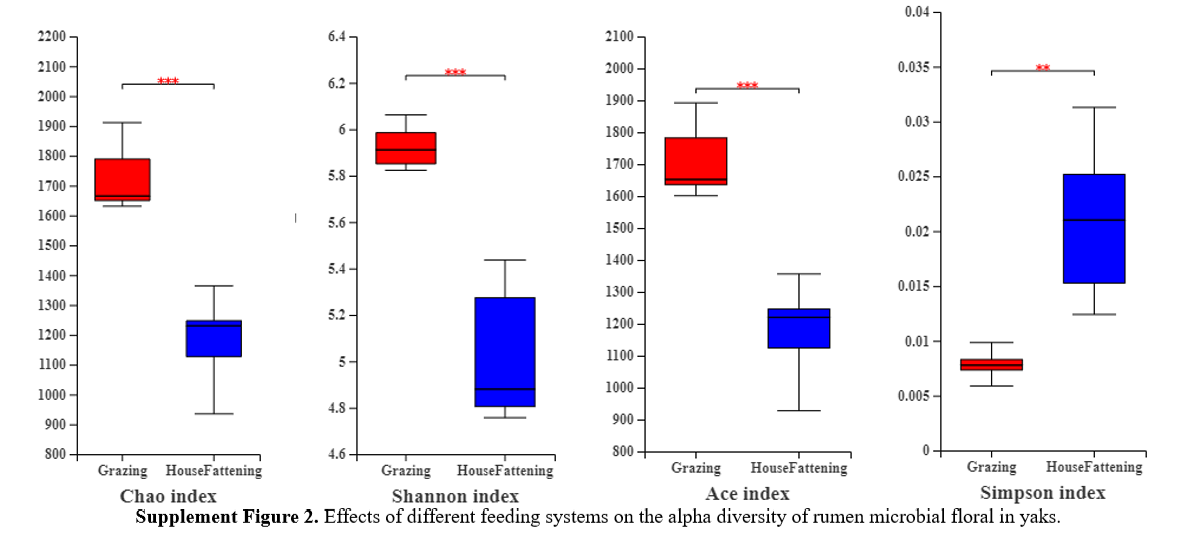

Supplement: Supplementary file 2 [file Image_2.TIF]

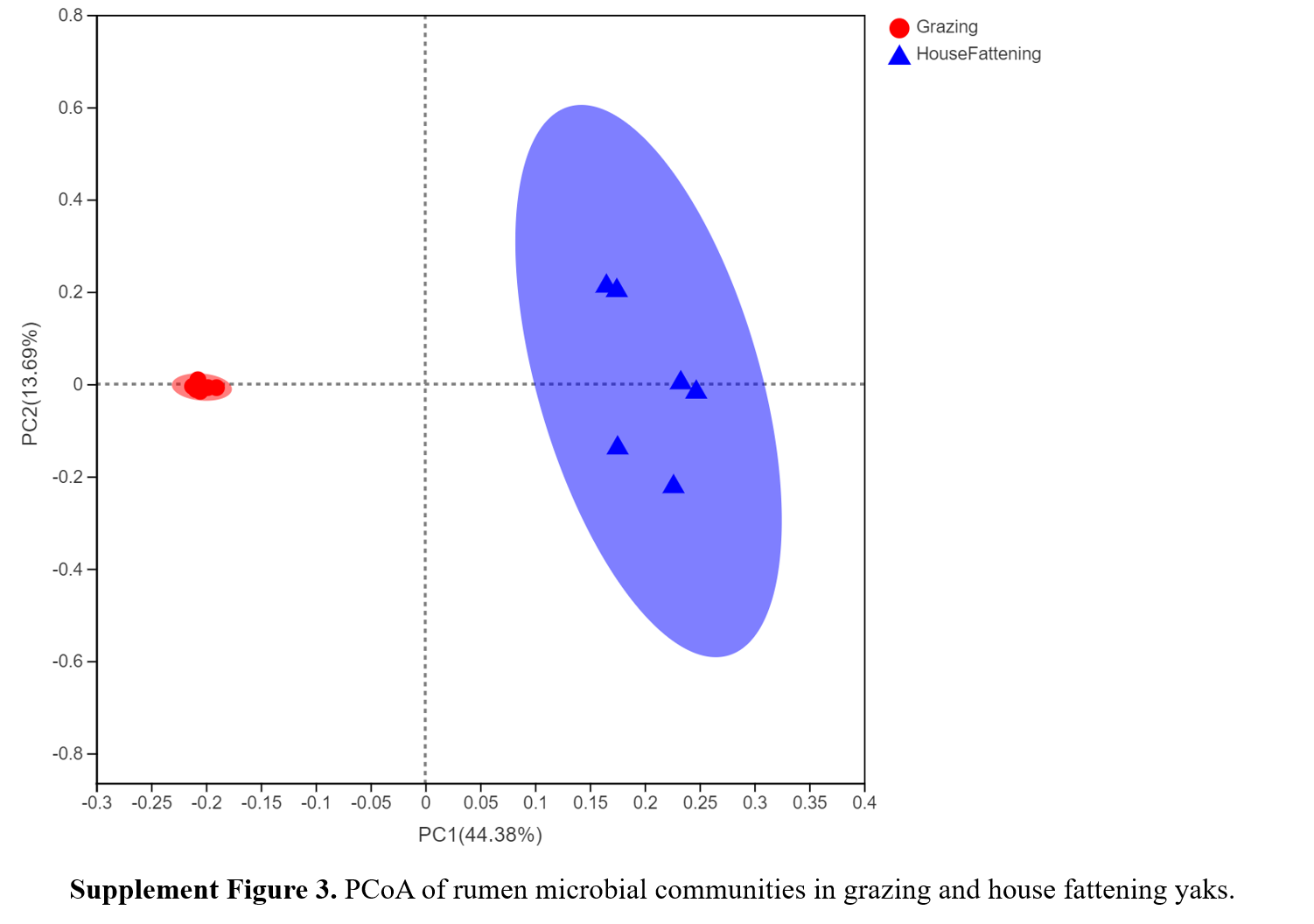

Supplement: Supplementary file 3 [file Image_3.TIF]
