# Supplementary material for: Microbiome and Metabolomics Reveal the Effects of Different Feeding Systems on the Growth and Ruminal Development of Yaks
Source: Front Microbiol. 2021 Jun 22;12:682989. doi: 10.3389/fmicb.2021.682989 (PMC8265505; doi:10.3389/fmicb.2021.682989)
Supplement: Supplementary file 4 [file Data_Sheet_1.PDF]

Supplement Table S1. Basic Diet Composition of Group HF

Item	Content(%)
Corn	48.53
Wheat Bran	6.22
Rapeseed Cake	7.25
Baking Soda	0.25
Dairy Salt	0.25
4% Premix Compound	1.5
Oat Hay	22
Alfalfa Hays	14
**Total**	**100**
