# Supplementary material for: Microbiome and Metabolomics Reveal the Effects of Different Feeding Systems on the Growth and Ruminal Development of Yaks
Source: Front Microbiol. 2021 Jun 22;12:682989. doi: 10.3389/fmicb.2021.682989 (PMC8265505; doi:10.3389/fmicb.2021.682989)
Supplement: Supplementary file 5 [file Data_Sheet_2.PDF]

Metabolite	VIP	RT(min)	M/Z	Fold change	P_value	Mode	Formula
**AMINO ACIDS, PEPTIDES, AND ANALOGS**							
2-Hepteneoylglycine	1.8975	2.66	184.10	0.02	0.0000	ESI-	$C_9H_{15}NO_3$
Glabrin C	3.5398	10.01	847.44	36.43	0.0002	ESI-	$C_{41}H_{64}N_8O_9$
Dopaquinone	2.1162	4.02	435.11	0.00	0.0000	ESI-	$C_9H_9NO_4$
Ecgoninium Methyl Ester(1+)	1.1136	3.05	445.26	164.63	0.0126	ESI-	$C_{10}H_{18}NO_3+$
Acetyl-DL-Leucine	1.5278	2.52	172.10	3.32	0.0000	ESI-	$C_8H_{15}NO_3$
Indolylacryloylglycine	3.6562	2.19	225.07	76.33	0.0000	ESI-	$C_{13}H_{12}N_2O_3$
Hydroxyprolyl-Proline	1.8424	1.31	273.11	47.55	0.0032	ESI-	$C_{10}H_{16}N_2O_4$
L-Tyrosine	1.8405	0.92	182.08	1.99	0.0447	ESI+	$C_9H_{11}NO_3$
D-Pipecolic acid	3.7453	0.70	130.09	0.43	0.0007	ESI+	$C_6H_{11}NO_2$
7-hydroxyoct-5-enoylglycine	1.534	1.65	198.11	3.79	0.0079	ESI+	$C_{10}H_{17}NO_4$
N-Palmitoyl GABA	4.638	10.75	721.55	10.33	0.0287	ESI+	$C_{20}H_{39}NO_3$
VPGPR Enterostatin	1.6308	6.34	569.27	0.12	0.0004	ESI+	$C_{23}H_{40}N_8O_6$
Tyrosyl-Proline	1.0347	1.85	311.16	2.06	0.0017	ESI+	$C_{14}H_{18}N_2O_4$
3-nonenoylglycine	2.5495	0.85	252.10	51.74	0.0003	ESI+	$C_{11}H_{19}NO_3$
**FATTY ACIDS AND CONJUGATES**							
Polyethylene, oxidized	2.9886	2.42	243.12	2.03	0.0044	ESI-	$C_{12}H_{20}O_5$
2,4-Dimethylpimelic acid	15.563	3.32	187.10	2.41	0.0000	ESI-	$C_9H_{16}O_4$
2-Ethylsuberic acid	9.3332	3.98	201.11	2.23	0.0009	ESI-	$C_{10}H_{18}O_4$
Xi-7-Hydroxyhexadecanedioic acid	7.4928	4.94	301.20	0.37	0.0000	ESI-	$C_{16}H_{30}O_5$
Suberic acid	3.1886	2.64	173.08	0.70	0.0117	ESI-	$C_8H_{14}O_4$
Hexadecanedioic acid	4.6745	6.89	285.21	0.14	0.0001	ESI-	$C_{16}H_{30}O_4$
Corchorifatty acid F	2.1762	5.72	327.22	0.68	0.0090	ESI-	$C_{18}H_{32}O_5$
(9Z,11R,12S,13S,15Z)-12,13-Epoxy-11-hydroxy-9,15-octadecadienoic acid	1.141	6.00	309.21	1.96	0.0081	ESI-	$C_{18}H_{30}O_4$
(+)-15,16-Dihydroxyoctadecanoic acid	4.0201	6.28	315.25	5.04	0.0000	ESI-	$C_{18}H_{36}O_4$
11-Hydroxy-9-tridecenoic acid	1.6652	4.09	273.17	1.44	0.0436	ESI-	$C_{13}H_{24}O_3$
Trans-2-trans-4-Heptadien-1-ol	2.7533	4.35	269.18	0.24	0.0001	ESI-	$C_7H_{12}O$
(S)-10,16-Dihydroxyhexadecanoic acid	1.8034	4.89	287.22	20.19	0.0003	ESI-	$C_{16}H_{32}O_4$
13,14-Dihydro PGF-1a	2.3978	4.89	403.27	17.31	0.0001	ESI-	$C_{20}H_{38}O_5$
Corchorifatty acid D	1.5187	4.93	307.19	2.20	0.0008	ESI-	$C_{18}H_{28}O_4$
9,10,13-Trihydroxystearic acid	2.4283	5.34	331.25	6.43	0.0442	ESI-	$C_{18}H_{36}O_5$
12-hydroxyheptadecanoic acid	1.649	5.91	331.25	0.53	0.0321	ESI-	$C_{17}H_{34}O_3$
Goshuyic acid	1.7693	6.12	269.18	7.80	0.0011	ESI-	$C_{14}H_{24}O_2$
5,7-Megastigmadien-9-ol glucoside	1.0816	6.58	337.21	0.68	0.0264	ESI-	$C_{19}H_{32}O_6$
4-Hydroxy-6-nonadecanone	1.0669	6.70	343.29	10.88	0.0000	ESI-	$C_{19}H_{38}O_2$
Reticulatamol	2.6007	10.27	579.50	5.83	0.0009	ESI-	$C_{35}H_{66}O_3$
28-Methyl-27-nonacosenoic acid	1.5383	10.29	495.44	13.61	0.0001	ESI-	$C_{30}H_{58}O_2$
8-Oxohexadecanoic acid	1.8977	9.39	539.43	6.14	0.0001	ESI-	$C_{16}H_{30}O_3$
12S-HHT	1.5258	9.11	605.41	0.22	0.0055	ESI-	$C_{17}H_{28}O_3$
2-hydroxyhexadecanoic acid	2.552	8.69	271.23	3.12	0.0010	ESI-	$C_{16}H_{32}O_3$
6S,9R-Dihydroxy-4,7E-megastigmadien-3	2.865	7.24	563.23	0.25	0.0000	ESI-	$C_{24}H_{38}O_{12}$
13,14-Dihydro-15-keto-PGE2	1.6118	6.82	351.22	0.53	0.0060	ESI-	$C_{20}H_{32}O_5$

3-Oxotetradecanoic acid	1.0705	5.65	287.19	10.46	0.0002	ESI-	$C_{14}H_{26}O_3$
11-Oxohexadecanoic acid	1.2223	5.32	315.22	1.90	0.0037	ESI-	$C_{16}H_{30}O_3$
9,10,13-TriHOME	4.067	5.28	329.23	7.50	0.0044	ESI-	$C_{18}H_{34}O_5$
Sorbitan laurate	1.9348	4.16	327.22	1.39	0.0050	ESI-	$C_{18}H_{34}O_6$
Methyl (R)-9-hydroxy-10-undecene-5,7-diynoate glucoside	2.0564	3.32	413.15	5.03	0.0000	ESI-	$C_{18}H_{24}O_8$
Undecanedioic acid	1.6849	2.73	261.13	3.02	0.0011	ESI-	$C_{11}H_{20}O_4$
Cucurbic acid	2.8891	2.45	257.14	13.05	0.0003	ESI-	$C_{12}H_{20}O_3$
Glutarate semialdehyde	1.1478	2.28	231.09	0.10	0.0047	ESI-	$C_5H_8O_3$
5-Acetamidovalerate	3.583	1.35	182.08	7.41	0.0014	ESI+	$C_7H_{13}NO_3$
10,20-Dihydroxyeicosanoic acid	2.1429	5.26	309.28	27.17	0.0071	ESI+	$C_{20}H_{40}O_4$
Geranyl tiglate	2.7532	4.96	237.18	0.19	0.0000	ESI+	$C_{15}H_{24}O_2$
Octadecanedioic acid	1.1665	6.41	337.23	6.61	0.0307	ESI+	$C_{18}H_{34}O_4$
7,8-Dihydrovomifoliol 9-[rhamnosyl-(1->6)-glucoside]	21.0936	8.70	535.27	0.28	0.0000	ESI+	$C_{25}H_{42}O_{12}$
Hexadecanedioic acid mono-L-carnitine ester	1.7863	5.92	474.28	0.48	0.0031	ESI+	$C_{23}H_{43}NO_6$
Cohibin C	3.4782	10.01	577.52	6.18	0.0056	ESI+	$C_{37}H_{68}O_4$
9-OxoODE	2.1996	5.74	295.23	2.76	0.0210	ESI+	$C_{18}H_{30}O_3$
**PURINES AND PURINE DERIVATIVES**							
Xanthine	3.837	0.73	151.02	1.26	0.0133	ESI-	$C_5H_4N_4O_2$
Adenine	5.3315	0.72	134.05	0.42	0.0003	ESI-	$C_5H_5N_5$
**NUCLEOSIDES, NUCLEOTIDES, AND ANALOGS**							
Adenosine	5.7687	0.88	312.09	0.08	0.0000	ESI-	$C_{10}H_{13}N_5O_4$
Guanosine	1.3585	0.88	282.08	0.26	0.0001	ESI-	$C_{10}H_{13}N_5O_5$
Xanthosine	2.058	0.99	283.07	0.35	0.0000	ESI-	$C_{10}H_{12}N_4O_6$
Inosine	4.6493	0.75	267.07	0.16	0.0003	ESI-	$C_{10}H_{12}N_4O_5$
Deoxyguanosine	1.8273	0.94	306.06	0.36	0.0005	ESI+	$C_{10}H_{13}N_5O_4$
7-Methylinosine	2.1137	2.63	347.12	0.19	0.0000	ESI+	$C_{11}H_{15}N_4O_5+$
2'-O-Methyladenosine	1.2979	1.16	282.12	0.19	0.0000	ESI+	$C_{11}H_{15}N_5O_4$
2'-Deoxyuridine	1.8104	0.89	227.07	1.79	0.0110	ESI-	$C_9H_{12}N_2O_5$
DUDP	2.1168	0.55	387.00	0.30	0.0087	ESI-	$C_9H_{14}N_2O_{11}P_2$
**OTHER LIPIDS AND LIPID-LIKE MOLECULES**							
MG(0:0/20:3(8Z,11Z,14Z)/0:0)	1.6172	5.88	425.29	0.08	0.0010	ESI-	$C_{23}H_{40}O_4$
Corchorifatty acid F	2.1762	5.72	327.22	0.68	0.0090	ESI-	$C_{18}H_{32}O_5$
1,2-Di-O-palmitoyl-3-O-(6-sulfoquinovopyranosyl)glycerol	3.5091	8.99	815.50	0.29	0.0000	ESI-	$C_{41}H_{78}O_{12}S$
MG(0:0/16:1(9Z)/0:0)	1.1154	5.49	373.26	0.29	0.0007	ESI-	$C_{19}H_{36}O_4$
(3R)-3,4-Dihydroxy-3-(hydroxymethyl)butanenitrile 4-glucoside	2.8228	0.92	316.10	4.48	0.0062	ESI+	$C_{11}H_{19}NO_8$
TG(14:0/20:4(8Z,11Z,14Z,17Z)/18:0)	4.1258	13.94	896.77	1.70	0.0007	ESI+	$C_{55}H_{98}O_6$
PE(15:0/20:0)	3.9087	11.37	778.56	6.46	0.0327	ESI-	$C_{40}H_{80}NO_8P$
1-(11Z-eicosenoyl)-glycero-3-phosphate	1.0338	7.42	509.29	2.41	0.0120	ESI-	$C_{23}H_{45}O_7P$
LysoPE(18:2(9Z,12Z)/0:0)	1.2347	7.85	476.28	2.43	0.0135	ESI-	$C_{23}H_{44}NO_7P$
1-Heptadecanoylglycerophosphoethanolamine	1.1941	8.53	466.29	0.54	0.0037	ESI-	$C_{22}H_{46}NO_7P$

PI(16:0/16:0)	3.0232	9.23	831.50	0.24	0.0000	ESI-	$C_{41}H_{79}O_{13}P$
PG(18:1(11Z)/18:1(11Z))	2.0542	9.98	773.53	6.70	0.0001	ESI-	$C_{42}H_{79}O_{10}P$
PE(15:0/18:2(9Z,12Z))	2.715	10.50	700.49	2.11	0.0000	ESI-	$C_{38}H_{72}NO_8P$
PG(18:0/20:3(5Z,8Z,11Z))	1.3876	10.62	781.54	13.68	0.0034	ESI-	$C_{44}H_{81}O_{10}P$
PE(16:0/18:1(11Z))	10.416	11.15	716.52	3.93	0.0000	ESI-	$C_{39}H_{76}NO_8P$
PE(18:1(11Z)/P-16:0)	2.4969	11.53	700.53	0.64	0.0361	ESI-	$C_{39}H_{76}NO_7P$
PE(18:0/18:1(11Z))	1.8166	11.75	744.55	1.84	0.0173	ESI-	$C_{41}H_{80}NO_8P$
PE(16:0/P-16:0)	3.0565	11.45	674.51	0.30	0.0000	ESI-	$C_{37}H_{74}NO_7P$
PE(15:0/P-16:0)	3.2231	11.15	660.50	0.52	0.0003	ESI-	$C_{36}H_{72}NO_7P$
PI(16:0/18:2(9Z,12Z))	2.8386	9.42	833.52	1.95	0.0017	ESI-	$C_{43}H_{79}O_{13}P$
LysoPE(0:0/16:1(9Z))	1.132	7.84	496.27	6.81	0.0155	ESI-	$C_{21}H_{42}NO_7P$
Lucidenic acid H	1.6958	3.07	457.26	709.94	0.0299	ESI-	$C_{27}H_{40}O_7$
Lucidenic acid N	6.139	3.12	459.27	85.14	0.0202	ESI-	$C_{27}H_{40}O_6$
Kanokoside D	2	8.26	623.25	0.14	0.0008	ESI-	$C_{27}H_{44}O_{16}$
Dehydrocyanaropicrin	1.0439	3.07	389.12	1.50	0.0107	ESI-	$C_{19}H_{20}O_6$
4,5-Dihydrovomifoliol	1.1514	4.51	271.15	1.81	0.0027	ESI-	$C_{13}H_{22}O_3$
Medicagenic acid	2.7156	6.66	501.32	55.34	0.0077	ESI-	$C_{30}H_{46}O_6$
Melleolide	1.3612	9.34	381.17	2.83	0.0164	ESI-	$C_{23}H_{28}O_6$
7,8-Dehydro-beta-micropteroxanthin	2.7563	10.63	833.57	3733.69	0.0238	ESI-	$C_{27}H_{38}O_2$
(2Z,6E)-3,7,11,15,19-Pentamethyl-2,6-eicosadien-1-ol	1.631	10.30	409.37	0.40	0.0079	ESI-	$C_{25}H_{48}O$
Sonchifolin	1.7542	9.26	355.16	2.62	0.0094	ESI-	$C_{21}H_{26}O_6$
Asperagenin	2.3802	8.06	429.30	4.84	0.0078	ESI-	$C_{27}H_{44}O_5$
Gamma-Tocotrienol	2.1887	6.86	455.32	12.33	0.0000	ESI-	$C_{28}H_{42}O_2$
Hovenidulcigenin A	1.4854	6.56	543.33	2.56	0.0002	ESI-	$C_{32}H_{48}O_7$
3-hydroxypristanic acid	1.1259	4.58	359.28	0.51	0.0134	ESI-	$C_{19}H_{38}O_3$
Ichangin 4-glucoside	2.0931	2.24	695.26	225.87	0.0000	ESI-	$C_{32}H_{42}O_{14}$
Melledonal A	1.0605	1.27	477.17	2.00	0.0027	ESI-	$C_{23}H_{28}O_8$
3-Hydroxy-beta-ionone	1.0571	3.69	209.15	0.36	0.0022	ESI+	$C_{13}H_{20}O_2$
Sclareol	1.7002	6.42	291.27	56.59	0.0046	ESI+	$C_{20}H_{36}O_2$
1-Aminocyclohexanecarboxylic acid	2.53	0.70	144.10	4.14	0.0288	ESI+	$C_7H_{13}NO_2$
Cynaratriol	1.3573	1.60	346.16	12.17	0.0032	ESI+	$C_{15}H_{22}O_5$
Crocin 3	2.1001	2.02	653.28	268.48	0.0112	ESI+	$C_{32}H_{44}O_{14}$
Ansamitocin P3	1.2263	3.10	635.27	7.32	0.0190	ESI+	$C_{32}H_{43}ClN_2O_9$
13'-Hydroxy-gamma-tocotrienol	2.0324	7.80	409.31	189.98	0.0011	ESI+	$C_{28}H_{42}O_3$
Ganoderic acid F	1.2709	8.45	593.27	0.19	0.0115	ESI+	$C_{32}H_{42}O_9$
3,6-Epoxy-5,5',6,6'-tetrahydro-b,b-carotene-3',5,5',6'-tetrol	2.7735	8.99	583.41	0.01	0.0000	ESI+	$C_{40}H_{58}O_5$
Ganoderenic acid E	2.2443	6.89	551.26	0.10	0.0000	ESI+	$C_{30}H_{40}O_8$
Cer(d18:0/14:0)	1.9465	10.49	556.49	1.83	0.0306	ESI-	$C_{32}H_{65}NO_3$
CerP(d18:1/12:0)	2.3616	9.96	606.41	24.20	0.0002	ESI-	$C_{30}H_{60}NO_6P$
CerP(d18:1/18:0)	3.7687	11.23	690.51	2.23	0.0066	ESI-	$C_{36}H_{72}NO_6P$
CE(17:0)	4.2627	11.30	675.54	1.75	0.0292	ESI-	$C_{44}H_{78}O_2$
Hebevinoside XII	2.0998	11.30	775.47	1.93	0.0083	ESI-	$C_{43}H_{70}O_{13}$

(3beta,5alpha,9alpha,22E,24R)-3,5,9-Trihydroxy-23-methylergosta-7,22-dien-6-one	2.41	8.34	439.32	20.44	0.0001	ESI-	$C_{29}H_{46}O_4$
Tetrahydrogestrinone	4.9504	8.29	669.42	394.75	0.0001	ESI-	$C_{21}H_{28}O_2$
3a,20b-Pregnanediol	1.295	7.28	365.27	0.06	0.0007	ESI-	$C_{21}H_{36}O_2$
Physapubescin	2.2026	6.88	553.28	0.20	0.0000	ESI+	$C_{30}H_{42}O_8$
Withangulatin A	1.24	8.00	549.25	0.39	0.0021	ESI+	$C_{30}H_{38}O_8$
Physangulide	1.7101	8.29	567.26	0.25	0.0083	ESI+	$C_{28}H_{42}O_9$
Digoxigenin monodigitoxoside	2.0779	8.38	565.28	0.11	0.0000	ESI+	$C_{29}H_{44}O_8$
(6alpha,22E)-6-Hydroxy-4,7,22-ergostatrien-3-one	2.0017	7.75	411.33	12.60	0.0011	ESI+	$C_{28}H_{42}O_2$
Physagulin C	5.671	7.24	565.24	0.17	0.0000	ESI+	$C_{30}H_{38}O_9$
**Others**							
Pantothenic Acid	4.1471	1.39	220.12	2.47	0.0147	ESI+	$C_9H_{17}NO_5$
Spirolide C	9.046	8.29	688.46	140.26	0.0019	ESI+	$C_{43}H_{63}NO_7$
Imidazolepropionic acid	1.1565	0.72	139.05	18.42	0.0028	ESI-	$C_6H_8N_2O_2$
Gentisic acid	4.6671	2.24	153.02	1.77	0.0356	ESI-	$C_7H_6O_4$
Hippuric acid	1.6845	1.96	178.05	0.25	0.0001	ESI-	$C_9H_9NO_3$
5-Phenyl-1,3-oxazinane-2,4-dione	4.824	3.21	226.03	0.30	0.0015	ESI-	$C_{10}H_9NO_3$
Vanilloylglycine	2.5181	1.84	206.05	28.81	0.0241	ESI-	$C_{10}H_{11}NO_5$
6-Methoxymellein	1.078	2.82	253.07	0.01	0.0214	ESI-	$C_{11}H_{12}O_4$
4-Acetyl-2(3H)-benzoxazolone	1.4234	1.27	222.04	40.60	0.0143	ESI-	$C_9H_7NO_3$
Apterin	1.398	3.65	459.11	0.00	0.0019	ESI-	$C_{20}H_{24}O_{10}$
Chalepin acetate	1.0522	4.46	377.14	0.03	0.0001	ESI-	$C_{21}H_{24}O_5$
Hydralazine pyruvate hydrazone	5.3635	2.00	229.07	0.02	0.0065	ESI-	$C_{11}H_{10}N_4O_2$
Erythratine	1.9278	1.70	316.15	5002.23	0.0028	ESI+	$C_{18}H_{21}NO_4$
5,7-Dihydroxy-4'-methoxy-8-methylflavanone	3.2091	2.10	345.10	0.21	0.0000	ESI-	$C_{17}H_{16}O_5$
Heliannone B	1.4608	2.90	345.10	0.35	0.0018	ESI-	$C_{17}H_{16}O_5$
Puddumin A	1.24	3.34	483.11	0.11	0.0143	ESI-	$C_{22}H_{24}O_{10}$
Apigenin 7-sulfate	1.0797	4.49	349.00	0.03	0.0001	ESI-	$C_{15}H_{10}O_8S$
Cycloartocarpin	1.0281	3.50	479.17	0.15	0.0000	ESI-	$C_{26}H_{26}O_6$
(3''-Apiosyl-6''-malonyl)astragalin	1.7367	4.43	708.18	0.05	0.0000	ESI+	$C_{29}H_{30}O_{18}$
6'-Hydroxyenterolactone	1.833	3.22	313.11	0.56	0.0021	ESI-	$C_{18}H_{18}O_5$
Fragransin D3	3.3151	3.76	409.16	0.01	0.0000	ESI-	$C_{22}H_{28}O_6$
2-Hydroxyenterolactone	2.0672	4.19	313.11	0.15	0.0000	ESI-	$C_{18}H_{18}O_5$
Flazine	2.6947	3.55	309.09	286.45	0.0020	ESI+	$C_{17}H_{12}N_2O_4$
1,4-Ipomeadiol	4.1642	2.55	215.09	14.28	0.0000	ESI-	$C_9H_{14}O_3$
3-Hydroxydodecanedioic acid	1.0708	3.38	283.10	0.59	0.0000	ESI-	$C_{12}H_{22}O_5$
Oxindole	1.8234	2.52	134.06	0.12	0.0001	ESI+	C_8H_7NO
5,6-Dihydroyangonin	1.3993	3.21	241.09	0.29	0.0000	ESI-	$C_{15}H_{16}O_4$
2-Carboxy-4-dodecanolide	1.63	5.28	241.14	1.95	0.0112	ESI-	$C_{13}H_{22}O_4$
Acetylcholine	2.0368	0.67	146.12	3.14	0.0017	ESI+	$C_7H_{15}NO_2$
Maltose	3.9012	0.62	341.11	22.03	0.0027	ESI-	$C_{12}H_{22}O_{11}$
N-Acetylgalactosamine	1.35	1.04	202.07	4.16	0.0000	ESI-	$C_8H_{15}NO_6$
Adlupone	1.7913	9.04	481.34	5.97	0.0093	ESI-	$C_{31}H_{46}O_4$

2-Methyl-3-nonacosanone	1.2313	12.18	481.46	2.96	0.0027	ESI-	$C_{30}H_{60}O$
Erucoylacetone	1.8041	10.14	423.35	0.29	0.0004	ESI-	$C_{25}H_{46}O_2$
Melibiose	3.4458	0.60	377.09	41.39	0.0053	ESI-	$C_{12}H_{22}O_{11}$
Doxorubicin-semiquinone	3.2636	3.24	512.19	0.49	0.0027	ESI+	$C_{27}H_{33}NO_{11}$
(1S,2S,4S,5R)-1,8-Epoxy-p-menthane-2,5-diol	1.2016	3.16	231.12	4.97	0.0008	ESI-	$C_{10}H_{18}O_3$
5-(3',5')-Dihydroxyphenyl-gamma-valerolactone	1.8726	3.02	255.09	0.00	0.0440	ESI-	$C_{11}H_{14}O_4$
Homovanillin	1.0193	1.72	211.06	0.22	0.0000	ESI-	$C_9H_{10}O_3$
(-)-Epinephrine	1.1675	1.40	184.10	4.31	0.0030	ESI+	$C_9H_{13}NO_3$
Dihydro-3-coumaric acid	5.8301	2.97	165.05	0.09	0.0000	ESI-	$C_9H_{10}O_3$
Hydroxyphenyllactic acid	1.2893	1.60	181.05	2.33	0.0000	ESI-	$C_9H_{10}O_4$
1-Hydroxy-6-methoxypyrene	1.1559	3.06	249.09	0.06	0.0002	ESI+	$C_{17}H_{12}O_2$
4-Pyridoxic acid	1.3572	1.39	228.05	8.55	0.0000	ESI-	$C_8H_9NO_4$
3-Pyridinebutanoic acid	2.4897	2.99	148.08	15.07	0.0017	ESI+	$C_9H_{11}NO_2$
(Z)-Resveratrol 3-(3''-sulfoglucoside)	1.3607	1.60	469.08	2867.29	0.0000	ESI-	$C_{20}H_{22}O_{11}S$
(E)-Squamosamide	1.5494	3.86	510.18	0.12	0.0000	ESI-	$C_{26}H_{27}NO_7$
D-Urobilin	1.9584	7.58	611.29	0.16	0.0101	ESI+	$C_{33}H_{40}N_4O_6$
Calystegin A3	1.3541	1.13	160.10	3.47	0.0107	ESI+	$C_7H_{13}NO_3$
